# Community-Based HIV-1 Early Diagnosis and Risk Behavior Analysis of Men Having Sex with Men in Hong Kong

**DOI:** 10.1371/journal.pone.0125715

**Published:** 2015-04-27

**Authors:** Jianguo Liang, Li Liu, Mandy Cheung, Man-Po Lee, Haibo Wang, Chun-ho Li, Chun-Chung Chan, Kenji Nishiura, Xian Tang, Zhiwu Tan, Jie Peng, Ka-Wai Cheung, Wing-Cheong Yam, Zhiwei Chen

**Affiliations:** 1 AIDS Institute and Department of Microbiology, Research Center for Infection and Immunity, Li Ka Shing Faculty of Medicine, The University of Hong Kong, 21 Sassoon Road, Pokfulam, Hong Kong Special Administrative Region, China; 2 AIDS Concern, 17B, Block F, 3 Lok Man Road, Chai Wan, Hong Kong Special Administrative Region, China; 3 Department of Medicine, Queen Elizabeth Hospital Hong Kong, Hong Kong Special Administrative Region, China; 4 Department of Microbiology, Queen Mary Hospital, the University of Hong Kong, Hong Kong Special Administrative Region, China; David Geffen School of Medicine at UCLA, UNITED STATES

## Abstract

The increasing prevalence of HIV-1 among men having sex with men (MSM) calls for an investigation of HIV-1 prevalence and incidence in MSM by early diagnosis to assist with early preventive interventions in Hong Kong. The participants were recruited randomly from MSM communities within a one-year period. Rapid HIV Test (RHT) and real-time dried blood spot (DBS)-based quantitative polymerase chain reaction (DBS-qPCR) were used for the early diagnosis of 474 participants. Risk behavior analysis was performed by studying information obtained from the participants during the study period. The HIV-1 prevalence and incident rates in the studied MSM population were 4.01% (19/474) and 1.47% (7/474), respectively. Three infected participants were found at the acute phase of infection by DBS-qPCR. Only 46.4% (220/474) MSM were using condoms regularly for anal sex. HIV infection significantly correlated with unprotected receptive anal sex and syphilis infection. An increased number of infections was found among foreign MSM in Hong Kong. This study is the first to use DBS-qPCR to identify acutely infected individuals in a community setting and to provide both the prevalence and incident rates of HIV-1 infection among MSM in Hong Kong. The risk analysis provided evidence that behavior intervention strengthening is necessary to fight against the increasing HIV-1 epidemic among MSM in Hong Kong and surrounding regions in Asia.

## Introduction

In 2014, the number of HIV-1 infections reached a historically high level in Hong Kong, with 195 new cases identified in the third quarter of the year, bringing the cumulative total of reported HIV infections to 6841 since 1984, when the first case of HIV was reported. Of the 195 HIV cases reported in the third quarter, 53.8% (105/195) acquired the infection via homosexual or bisexual contact, 17.9% (35/195) via heterosexual contact, and one via drug injection[1]. In fact, MSM have become the dominant risk group of the increasing HIV-1 prevalence in Hong Kong in recent years. Although antiretroviral treatments (ARTs) and preventive interventions have been provided (e.g., education and free condom programs)[2–5], 67.6% of male HIV reports in 2013 contracted the virus through homosexual or bisexual contact [1]. Recent studies have suggested that HIV-1 subtype B and CRF01_AE variants are predominant in Hong Kong, with a growing number of subtype B transmission clusters among local MSM beginning in 2003[6, 7]. The alarming trend of MSM infections indicates the need to track sexually active and acutely infected individuals for targeted prevention. Thus far, no studies have investigated the incidence rate of MSM infections in Hong Kong over the 30-year period of the HIV-1 infection epidemic. For community settings, establishing a rapid diagnostic method to capture acutely infected individuals is necessary for early prevention intervention and treatment to avoid transmission in a timely fashion. This type of intervention is highly relevant to the clustering spread of local HIV-1 strains, particularly because this spread has been well documented among MSM groups in Hong Kong in recent years [8, 9].

Free HIV-1 diagnosis is readily available at sites offered by Department of Health or different NGOs. Currently, the most common diagnosis method for HIV-1 infection is the commercial Rapid HIV test (RHT) Despite the convenience, accuracy and cost-effectiveness of RHT, this method can only detect the HIV-1 specific antibody after a three-month “window period” [10, 11]. During this “window period”, acutely infected individuals with high viral load will enhance the probability of viral transmission[12]. For sexually active MSM, the virus can be transmitted from one person to multiple partners during this phase and result in the typical clustering spread that has been documented in Hong Kong [8, 9]. Therefore, exploring new approaches for early diagnosis is essential for reducing the viral transmission rate.

The nucleic acid-based test (NAT), which is capable of detecting one or more target sequences of HIV-specific genes, is used for the early diagnosis of HIV-1[13]. Although multiple commercial kits (bDNA, Roche Amplicor Ultrasensitive, and Cobas) are readily available for measuring HIV-1 viral load and for early diagnosis, these products are expensive, and the necessary equipment is inaccessible[14]. Additionally, venous blood draw for these assays is extremely difficult at community settings, including those being run by NGOs in Hong Kong. Therefore, dried blood spots (DBS) have been utilized for early HIV-1 detection in infants, viral load and drug resistance monitoring, as well as in places without facilities for sample processing or cold storage[14–16].

In this study, we aimed to establish a prompt, sensitive and specific real-time DBS-based quantitative PCR (DBS-qPCR) for rapid HIV diagnosis at the community level, which may potentially benefit early preventive interventions. We showed that early HIV-1 infection in MSM could be diagnosed by combining a DBS-based method and RHT in our local community settings. In addition, we provided evidence for strengthening the timely behavior and biomedical interventions for fighting against the HIV-1 epidemic in Hong Kong through determining the prevalence and incident rates of HIV-1 infection among MSM by risk analysis.

## Materials and Methods

Ethical approval was obtained from the Institutional Review Board of the University of Hong Kong/Hospital Authority Hong Kong West Cluster (UW09-434), and written informed consent was obtained from the study subjects before peripheral blood collection. This content procedure was approved by the ethics committee/IRBs.

### HIV-1 RHT

HIV-1 RHT was conducted on site using Determine HIV 1/2 test (Abbott Laboratory, North Chicago, Illinois, USA) by well-trained staff members at our community-based NGO settings. The results were analyzed and properly recorded according to the manufacturer’s instructions. In the meantime, a voluntary counseling and testing (VCT) interview was performed using a questionnaire collected confidentially from each participant.

### DBS collection

With proper informed consent, all DBS samples were collected by a local NGO, namely, AIDS Concern in Hong Kong. In total, 474 DBS samples were collected between March 2010 and April 2011 from sexually active high-risk MSM groups at local MSM venues, including bars, saunas, and clubs, as well as at VCT offices of AIDS Concern. Dried blood spots were collected by spotting five blood drop samples (50 μl each) onto each of five rings on a Whatman 903 paper (Whatman Inc., Piscataway, NJ, USA). After the spots were air-dried thoroughly at room temperature, each Whatman 903 paper was bar-coded and sealed in a plastic bag for DNA extraction.

### Risk factor interview

The risk factor interview was an investigator-based interview designed to examine the risk factors associated with HIV-1 infection. The variables collected (e.g., the channel to look for partners, sex partners, sexual behavior, role in anal sex, use of a condom, and sexually transmitted diseases) included the 3 months before blood collection.

### DNA extraction

Genomic DNA was isolated from the DBSs using a Qiagen microDNA extraction kit (Qiagen, Hilden, Germany). To extract DNA from the DBSs, three blood spot samples (3 mm diameter) from each participant were combined into a 1.5 ml micro-centrifuge tube. Then, 180 μl Buffer ATL and 20 μl proteinase K were added to the tube, followed by thorough vortex mixing of the tube. Next, the tube was incubated at 56°C in a heated block for 1 h with 10 s of vortexing every 10 min. After the tube was incubated, 200 μl Buffer AL was added to the mixture, and the tube was incubated at 70°C in a heated block for 10 min with 10 s of vortexing every 3 min. Subsequently, the entire lysate was transferred to a QIAamp MinElute column and centrifuged for 1 min. The column was opened carefully after centrifugation, and 500 μl each of Buffer AW1 and AW2 were added. Finally, the column was placed in a clean 1.5 ml micro-centrifuge tube, and DNA was eluted by applying 50 μl double distilled water to the center of the membrane. DNA was obtained after centrifuging the tube at 14,000 rpm for 1 min. The remaining two blood spot samples were stored properly (at either 4°C or -20°C) for use with later confirmation tests.

### Evaluation of DBS-derived DNA quality

The quality of DBS-derived DNA was evaluated by the detection of the human housekeeping gene Cyclophilin A (CycA) from the extracted DNA before use. The CycA gene was amplified from the extracted DNA by conventional reverse transcriptase PCR using the following primers: forward primer, 5’-CATCTGCACTGCCAAGACTGAGTG-3’, and reverse primer, 5’-CTTCTTGCTGGTCTTGCCATTCC-3’. The expression of the CycA gene was assured by gel electrophoresis after loading the PCR product into a 1% agarose gel.

### Evaluation of the sensitivity and linearity of the real-time assay

Real-time PCR was conducted in a single-tube system using a Stratagene Mx3000P Real-Time PCR Platform (Agilent Technologies, Santa Clara, CA). The plasmid of NL4-3 R^-^E^-^Luc was serially diluted for evaluating the sensitivity and linearity of real-time PCR (from 10^8^ to 10^10^ copies). The HIV-1 LTR gene from DBS-derived DNA was quantified by real-time PCR assay using the following primers: forward primer HIV LTR-506, 5’-GRAACCCACTGCTTAASSCTCAA-3’, and reverse primer HIV LTR-626, 5’-TGTTCGGGCGCCACTGCTAGAGA-3’ [14]. After the amplification cycles, melting curve analysis was performed to confirm the specificity of each amplified sample.

### Sensitivity of the DBS-qPCR assay for multiple HIV-1 subtypes

HIV-1 infected blood samples representing A/E, B and other prevalent HIV-1 clades in Hong Kong were obtained from our local AIDS clinics. The HIV-1 LTR gene from genomic DNA was quantified by real-time PCR using SYBR Green and the following primers: forward primer HIV LTR-506, 5’-GRAACCCACTGCTTAASSCTCAA-3’, and reverse primer HIV LTR-626, 5’-TGTTCGGGCGCCACTGCTAGAGA-3’ [14]. After the amplification cycles, melting curve analysis was performed to confirm the specificity of each sample. The plasmid of NL4-3 R-E-Luc was serially diluted for evaluating the sensitivity and linearity of the real-time PCR (from 10^8^ to 10^10^ copies).

### Sequencing of diagnosed HIV-1 samples

DNA extracted from HIV-1-positive samples that were confirmed by RHT or DBS-qPCR was subjected to a nested PCR using primers based on the HIV-1 pol region[17]. An approximately 400-bp pol fragment was amplified by PCR with nested primers using DNA polymerase (Invitrogen, USA). The following PCR primers were used: 5’-TCTCTCGACGCAGGACTCGGCTTG-3’ (sense) and 5’-TCTAATACTGTATCATCTGCTCCTG-3’ (antisense) for the first-round reaction, and 5’-TTTGACTAGCGGAGGCTAGAAG-3’ (sense) and 5’-GCTCCTTCTGATAATGCTGAAAACATGGG-3’ (antisense) for the second-round reaction. The amplification cycles were 95°C for 2 min, followed by 35 cycles of 95°C for 15 s, 55°C for 45 s, and 72°C for 30 s, with a final extension at 72°C for 1 min. The amplified PCR products were purified using a QIAquick PCR purification kit (Qiagen) and subjected directly to DNA sequencing using an automated ABI 377 DNA sequencer at Beijing Genomics Institute, Hong Kong.

### Phylogenetic analysis of HIV-1 sequences

To eliminate potential contamination, all of the obtained sequences were first subjected to an HIV-1 Blast search to compare these sequences with related reference sequences in the HIV Databases, which are funded by the Division of AIDS of the National Institute of Allergy and Infectious Diseases (NIAID), a part of the National Institutes of Health (NIH) (http://hiv-web.lanl.gov/content/index). Nucleotide sequences were aligned with the references using the ClustalX 1.81 program and further adjusted manually[18]. The genetic distances of the analyzed HIV-1 sequences were calculated using Kimura’s two parameter model [19]. Phylogenetic trees were generated using the neighbor-joining method as implemented in the ClustalX 1.81 program. The branch significance was analyzed by bootstrap with 1,000 replicates. The trees were printed using the TreeView program[20].

### Nucleotide sequence accession numbers

The GenBank accession numbers of the HIV-1 sequences reported here are under the submission code KP418566-KP418573‏.

### Statistical analysis

Pearson’s *x* ^2^ test and Fisher’s exact test were performed to investigate the differences in the demographic data. All statistical analyses were performed using the software IBM SPSS Statistics 19. A p value equal to 0.05 was considered significant, and Bonferroni adjustments were made when conducting multiple comparisons. Multivariate logistic regression analysis was conducted using risk factors that had significant differences according to Pearson’s *x* ^2^ test or Fisher’s exact test.

## Results

### Demographics of Study Subjects

In this study, 976 participants were interviewed at our community-based VCT sites for MSM. Only 507 (51.9%) participants were willing to contribute extra blood drops for the DBS-qPCR test; the refusal rate was 48%. Based on the inclusion procedure, 474 valid specimens were included in this study from March 2010 to April 2011 (S1 Fig). Most MSM participants were sexually active, with an age range from 21 to 40 years old and accounting for 72.4% (343/477) of all participants. Additionally, 77.2% of the sexually active MSM participants were Hong Kong residents (366/474). According to our data, only 24.5% of the participants claimed to have regular partners; 57.6% (273/474) of participants had 1 to 3 sex partners, while 29.1% (138/474) had more than 4 sex partners during the 3-month period before blood collection. The venues that participants used to seek their sex partners included bars/clubs, saunas, the Internet and friends, which account for 18.1% (86/474), 38.6% (183/474), 32.5% (154/474) and 44.3% (210/474), respectively. The participants engaged in multiple sexual behaviors, including 82.7% (392/474) anal sex, 81.0% (384/474) oral sex and 40.5% (190/474) mutual masturbation, whereas only 46.4% (220/474) of MSM used condoms regularly. These results indicated the complexity and dynamic nature of MSM in Hong Kong.

### In-house DBS-qPCR assay validation

To ensure the quality of the extracted DNA, we performed a PCR using primers for the human housekeeping gene Cyclophilin A (CycA) as described previously[21–24]. Only CycA positive samples were used for the DBS-qPCR assay. During the study period, only 3 samples did not have a sufficient amount of DNA due to the poor quality of blood collection on filter papers. Then, we evaluated the sensitivity and specificity of our in-house DBS-qPCR assay. The primers were designed based on a highly conserved region of HIV-1 LTR sequences that amplifies all major HIV-1 subtypes in the world[25]. Serially diluted pNL4-3 R-E-Luc DNA was used to evaluate the sensitivity and linearity of our qPCR (From 10^8^ to 10^10^ copies). About 50–100 copies of HIV-1 DNA were successfully detected with a 0.995 correlation coefficient. No non-specific amplicon was produced during amplification according to the dissociation curve analysis (data not shown). During the study, 30 HIV-1 patient blood samples were assayed for the presence of HIV-1 proviral DNA, and among these samples, 29 samples showed a positive result as detected by DBS-qPCR. This result demonstrated the competence ability of this method to detect multiple HIV-1 subtypes, as shown in Table 1. A 100% detection rate was found for subtype A/E and B groups, which present the most prevalent subtypes in Hong Kong. No HIV-1 proviral DNA was detected in the 2 blood samples of healthy individuals. These results confirmed the necessary sensitivity and specificity of the DBS-qPCR assay for practical application.

**Table 1:** HIV-1 genetic diversity and detection of pro-viral HIV-1 DNA using SYBR Green Real-Time PCR assays.

Subtype	Number	Proviral DNA	Risk factors			
		Detection by DBS-qPCR	Homosexual	Heterosexual	Bisexual	Blood
A/E	6	6/6 (100%)	6	0	0	0
B	14	14/14 (100%)	8	4	2	0
Other	2	2/2 (100%)	0	2	0	0
N/D	8	7/8 (87.5%)	7	0	0	1
Total	30	29/30 (96.7%)	21	6	2	1
N/D: not done						

### HIV-1 prevalence and incident rates of MSM in Hong Kong

**Fig 1:**
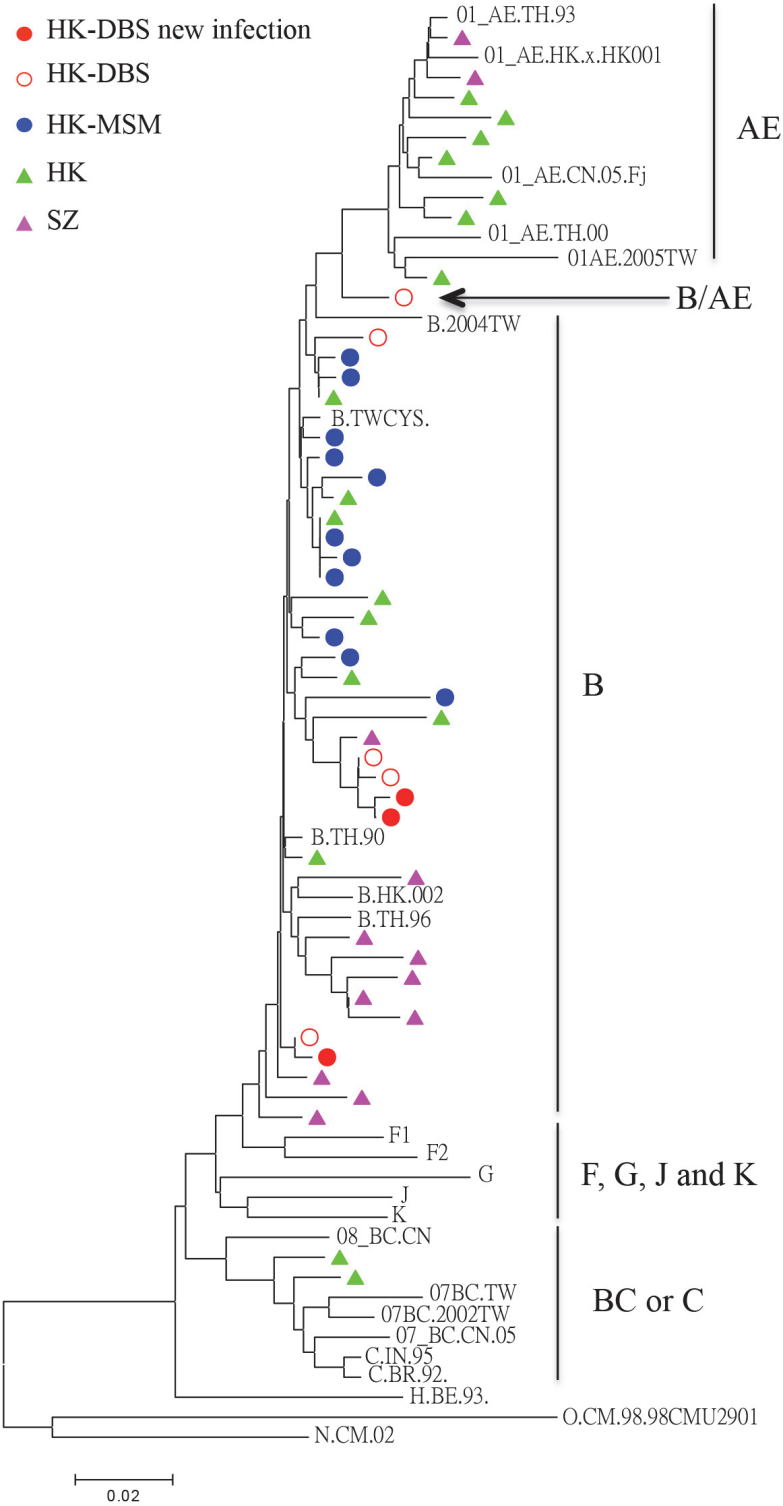
Phylogenetic neighbor-joining tree for HIV-1 pol sequences obtained from DBSs of MSM in Hong Kong. The reference sequences for classifying HIV-1 genotypes were included and were originally obtained from the NIH/NIAID–funded HIV Databases. The source of HIV-1 strains is color-coded. The horizontal branch was drawn in accordance with their relative genetic distances as indicated by the bar scale. The vertical lines are purely for clarity of the tree presentation.

In total, 474 specimens were assayed by both RHT and DBS-qPCR. Nineteen individuals were identified as HIV-1-positive in one of the assays (Table 2), which gave a HIV prevalence rate of 4.01% (19/474) among tested participants. Thirteen of 19 samples were determined to be HIV-1-positive by both assays, and 3 samples were determined to be positive by RHT but not by DBS-qPCR. This discrepancy is most likely due to insufficient DNA and low proviral copies. Four positive individuals from the 19 samples claimed to be negative for RHT as tested elsewhere approximately six months ago, suggesting new infections during our study period. Three other samples from the 19 samples were determined to be positive only by DBS-qPCR assay. These results were further confirmed by additional DBS tests and by sequencing analysis. The single positive result for DBS-qPCR assay but not for RHT in these 3 participants revealed acute infections before seroconversion. Therefore, a 1.47% (7/474) incidence rate was identified among the MSM study participants. Interestingly, phylogenetic analysis showed that sequences from these three acutely infected patients closely clustered with subtype B reference sequences. One of the sequences is closely related to subtype B sequences isolated from Hong Kong and other two are clustered with sequences from Shenzhen, China. These results suggest that these three acutely infected patients harbor strains of HIV-1 subtype, which is one of the predominant subtypes in the MSM population in Hong Kong and in the neighboring city Shenzhen (SZ) (Fig 1) **[8, 9, 26]**. Two of these three strains clustered closely, suggesting a possible transmission relationship. Moreover, one sequence was between the cluster of CRF01AE and subtype B, suggesting that this DBS-derived virus was likely a new recombinant strain of subtype B and CRF01AE, as indicated by an arrow in Fig 1.

**Table 2:** Comparison of DBS-qPCR and RHT test results.

Abbott RHT	DBS-qPCR		Total
	Positive	Negative	
Positive	13	3	16
Negative	3	455	458
Total	16	458	474

### Risk factors associated with MSM infections in Hong Kong

To elucidate the risk factors that may correlate with HIV-1 infection among the studied MSM population, a comprehensive analysis was conducted, as shown in Table 3. We found that the risk behavior of anal sex was significantly associated with HIV-1 infection (p = 0.009). In particular, the proportion with the insertive role was significantly lower among infected MSMs (p = 0.004) compared with control MSMs. Further multivariate logistic regression analysis showed that the risk factor of insertive role is not an independent predictive factor for HIV positivity (p = 0.081). Additionally, this analysis showed that foreign MSM had a relatively higher proportion among infected people compared with uninfected MSM (p = 0.048), although the number remained small. Of note, the overall percentage of regular condom use in MSM was 46.4% (220/474) for anal sex but 3.2% (15/474) for oral sex. Moreover, we investigated the effect of sexually transmitted diseases on MSM infections. Our results showed that HIV-1 infection in MSM was significantly associated with co-infection of syphilis (p<0.001) but not with chlamydia, gonorrhea or hepatitis B, as shown in Table 4.

**Table 3:** Risk factors associated with HIV-1 infection among MSM in Hong Kong.

	HIV-1 (+)	HIV-1 (-)	*p‡*	*p§*
	n = 19 (%)	n = 455 (%)		
**Age**			0.812	
<20	0(0)	33(7.3)		0.626
21–40	14(73.7)	329(72.3)		0.773
>40	3(15.8)	70(15.4)		0.746
Unknown	2(10.5)	23(5.1)		
**Nationality**			0.084	
Mainland China	1(5.3)	46(10.1)		1
Hong Kong	13(68.4)	353(77.6)		0.326
Foreign Countries	3(15.8)	19(4.2)		**0.048**
Unknown	2(10.5)	37(8.1)		
Channel*			0.538	
Bar/Club^æ^	6(31.6)	80(17.6)		0.129
Internet^æ^	4(21.1)	150(33.0)		0.337
Sauna^æ^	6(31.6)	177(38.9)		0.635
Friends^æ^	8(42.1)	202(44.4)		0.988
Others^æ^	1(5.3)	32(7.0)		1
Unknown	2(10.5)	24(5.3)		
Sex Partner*			0.919	
Regular Partner	6(31.6)	110(24.2)		0.449
Irregular Partner	7(36.8)	136(29.9)		0.259
Unknown	6(31.6)	209(45.9)		
Number of sex partners*			0.076	
0	4(21.1)	35(7.7)		0.05
1-Mar	10(52.6)	263(57.8)		0.874
>4	3(15.8)	135(29.7)		0.235
Unknown	2(10.5)	22(4.8)		
Sex behavior*			0.756	
Anal Sex	17(89.5)	375(82.4)		0.239
Others	0(0)	1(0.2)		1
Unknown	2(10.5)	29(6.4)		
Role in anal sex* ^**ø**^			**0.009**	
Receptive	9(47.4)	132(29.0)		0.096
Insertive	0(0)	114(25.1)		**0.004**
Dual	7(36.8)	123(27.0)		0.388
Unknown	3(15.8)	86(18.9)		
Anal sex with Condom*			0.85	
Never	3(15.8)	100(22.0)		0.773
Every Time	8(42.1)	212(46.6)		0.892
Sometimes	6(31.6)	123(27.0)		0.585
Unknown	2(10.5)	20(4.4)		
Oral sex with Condom*			0.68	
Never	14 (73.6)	343 (75.4)		0.754
Every Time	0	15 (3.3)		0.435
Sometimes	3 (15.8)	75 (16.5)		0.972
Unknown	2 (10.5)	22 (4.8)		

The unknown groups were excluded from the analysis.

*Within 3 months before blood collection.

^æ^Shared factors.

^‡^Comparison of distribution between HIV-1 positive versus HIV-1 negative in each subcategory. Chi-square test when all cell frequency>10, otherwise Fisher’s exact test was employed. Bold numbers indicate statistical significance.

^***§***^Comparison of distribution between HIV-1 positive versus HIV-1 negative in each subcategory. Chi-square test when all cell frequency>10, otherwise Fisher’s exact test was employed. Bold numbers indicate statistical significance.

^ø^Multivariate logistic regression analysis was performed; the results are reported in the text only.

**Table 4:** Sexually transmitted diseases (STDs) among MSM in Hong Kong from March 2010 to February 2011.

	HIV-1 (+)	HIV-1 (-)	
	n = 19 (%)	n = 455 (%)	*p‡*
**Syphilis**			**<0.001**
Positive	5(26.3)	10(2.2)	
Negative	12(63.2)	425(93.4)	
Unknown	2(10.5)	20(4.4)	
**Chlamydia**			0.356
Positive	1(5.3)	9(2.0)	
Negative	16(84.2)	374(82.2)	
Unknown	2(10.5)	72(15.8)	
**Gonorrhea**			
Positive	0(0)	0(0)	
Negative	17(89.5)	382(84.0)	
Unknown	2(10.5)	73(16.0)	
**Hepatitis B**			0.345
Positive	1(5.3)	2(0.4)	
Negative	14(73.7)	98(21.5)	
Unknown	4(21.1)	355(78.0)	

The unknown groups refer to the participants who had not been tested; these participants were excluded from the analysis.

‡Comparison of distribution between HIV-1 positive versus HIV-1 negative in each subcategory. Chi-square test when all cell frequency>10, otherwise Fisher’s exact test was employed. Bold numbers indicate statistical significance.

## Discussion

In this study, we established a novel approach for early HIV-1 diagnosis by combining our in-house DBS-qPCR assay and the commercial RHT. DBS-qPCR is sensitive and can detect HIV-1 proviral DNA to a level as low as 50–100 copies. Our in-house assay was designed to cover all major subtypes of the HIV-1 M group and was extremely successful for the detection of circulating clades, including CRF01_AE and B/B’ found in Hong Kong. Using DBS-qPCR assays and RHTs, we conducted a large cross-sectional examination of MSM at our local community-based settings over a one-year period. The results revealed a 4.01% prevalence rate and 1.47% incidence rate among MSM participants in Hong Kong, which have not been reported previously. The risk factors associated with MSM infection are primarily the receptive role of anal sex, foreign MSM and syphilis. Nevertheless, our results supported the feasibility of using DBS-qPCR assays and RHTs together to identify acutely infected individuals for early preventive interventions, such as treatment as prevention (TAP) at our community settings. The risk behavior analysis also elucidated new evidence to strengthen necessary preventive interventions to fight against the rising MSM HIV-1 epidemic in Hong Kong.

The DBS-qPCR assay is a reliable and cost-effective assay [14, 27, 28]. This detection sensitivity of 50–100 copies is determined primarily by the use of SYBR Green dye instead of Taqman probe, which could detect as low as 1–10 copies[14, 29]. Apart from sensitivity, the DBS-qPCR assay showed high specificity in our platform, as demonstrated in Table 1. For HIV-1 diagnosis in our community settings, the DBS-qPCR assay results were 81.3% in agreement with the RHT results. In addition, the DBS-qPCR assay was able to detect 84.2% (16/19) of RHT HIV-1-positive specimens. The reason that one of the three RHT HIV-1-positive specimens was not detected by the DBS-qPCR assay was due to the improper preparation of blood spots and due to an insufficient amount of DNA. Additionally, the low copy number of HIV-1 proviral DNA (<50 copies) of the other two specimens may have been a factor because the detection of the housekeeping gene remained positive. The increased genetic diversity of the HIV-1 sequence in Hong Kong could also account for the detection failure[9]. These negative results suggested that future studies should address these technical issues carefully. Interestingly, three HIV-1-positive MSM were identified by DBS-qPCR assay but not by RHT. This result indicated these participants remained in the “window period” before seroconversion[11]. Because these infected subjects were further confirmed by testing two additional dried blood spots samples using sequence analysis, we concluded that using a combination of DBS-qPCR and RHT assays to identify acutely infected individuals for early preventive interventions is feasible.

MSM remains one of the major risk groups for HIV-1 infection in Hong Kong. The result reveals a 4.01% prevalence rate and a 1.47% incidence rate, which elucidated the alarming situation of the HIV-1 epidemic among our local MSM communities. In general, the risk behavior of MSM in Hong Kong is complex. As shown in Table 3, these behaviors, which included 1) multiple sex partners, 2) multiple partnership sources, 3) irregular sexual partnerships, 4) mixed sexual routes/contacts, 5) a low condom usage rate, 6) predominance of local MSM, 7) foreign MSM, and 8) STD status, are consistent with findings described previously [2, 4, 30–32]. In particular, the receptive role of anal sex (p = 0.009), foreign MSM (p = 0.048) and syphilis infection (p<0.001) were significantly associated with increased proportions of HIV-1 infection, as shown in Tables 3 and 4. Because most MSM (82.7%, 392/474) participants participated in anal sex within 3 months before the testing, the proportion with the insertive role was also significantly lower among infected MSM (p = 0.004) compared with control MSM. Therefore, providing intense public education to enhance the awareness of risky sexual behaviors among MSM is necessary.

Effective TAP requires an enhanced effort of HIV-1 diagnosis for action. Mounting evidence has demonstrated the efficacy of TAP worldwide, which includes 98–100% efficacy of mother-to-child transmission prevention, a 96% efficacy of HIV-1 discordant couples and greater transmission reduction among MSM[26, 33–35]. Similarly, one of the most successful stories in China was the province-wide screening of HIV-1 infection among all people who conducted paid blood donation (PBD), followed by the banning of PBD and the treatment of all infected individuals in Henan [36, 37]. As a result, Henan Province is no longer a major epidemic region compared with other provinces, such as Guangxi and Yunnan [17, 38]. Evidently, seeking infected people, followed by sustained treatment in a timely fashion, can be an extremely effective way to delay the HIV-1 epidemic in developing countries, including China. Thus, our community-based early diagnosis method may be particularly useful to strengthen the TAP of sexually active MSM.

In this study, all three newly infected patients harbored strains of HIV-1 subtype B, which is one of the predominant subtypes in the MSM population in Hong Kong [8, 9, 26]. Three viruses were found in two transmission clusters in the phylogenetic tree, suggesting a local or regional origin of these newly transmitted viruses. Additionally, one DBS-derived virus was found to be the recombinant strain of subtype B and CRF01AE. In fact, this report is the first to identify such a chimeric subtype among the MSM population in Hong Kong. Because these acutely infected individuals may have high viral loads with higher probabilities of viral transmission[39], these individuals may become potential fast spreaders to foster the expansion of these viral clusters (Fig 1). This expansion is possible, particularly when these participants are sexually active, have multiple sexual partners in the three months before diagnosis, and seldom use condoms. This study has some limitations. For example, the small sample volume of the DBSs was insufficient for “gold standard” PCR method for the validation of the DBS-PCR assay [25], and for additional HIV incidence assays as suggested by the WHO guidelines. The rate of refusal (48%) among study subjects remains high and needs to be improved in future studies. Further technique improvement should also explore full-length HIV genome sequencing and cloning from DBS.

In conclusion, given the latest modification of the WHO guidelines for HIV-1 treatment, early diagnosis may facilitate the efforts of early patient treatment. In addition to TAP, ARTs should be initiated during the early stage of infection, which could lead to decreased mortality and fewer complications in HIV-infected patients [23]. ARTs may also lead to potential functional cures for some acutely infected people [40]. History sometimes repeats itself with tragic public consequences. Hong Kong has already experienced a large expansion of HIV heterosexual infection after the first wave of the MSM epidemic. Currently, Hong Kong is experiencing the second but much larger wave of the MSM epidemic[2]. Our findings may have significant implications in strengthening strategic plans to prevent the second expansion of HIV heterosexual infections in Hong Kong or in regions with similar situations worldwide.

## Supporting Information

S1 Fig CONSORT diagram. MSM participants screened, enrolled and included in the Hong Kong analysis, March 2010 to February 2011.
(TIF)
