# Supplementary figures and images for: Community-Based HIV-1 Early Diagnosis and Risk Behavior Analysis of Men Having Sex with Men in Hong Kong
Source: PLoS One. 2015 Apr 27;10(4):e0125715. doi: 10.1371/journal.pone.0125715 (PMC4410921; doi:10.1371/journal.pone.0125715)

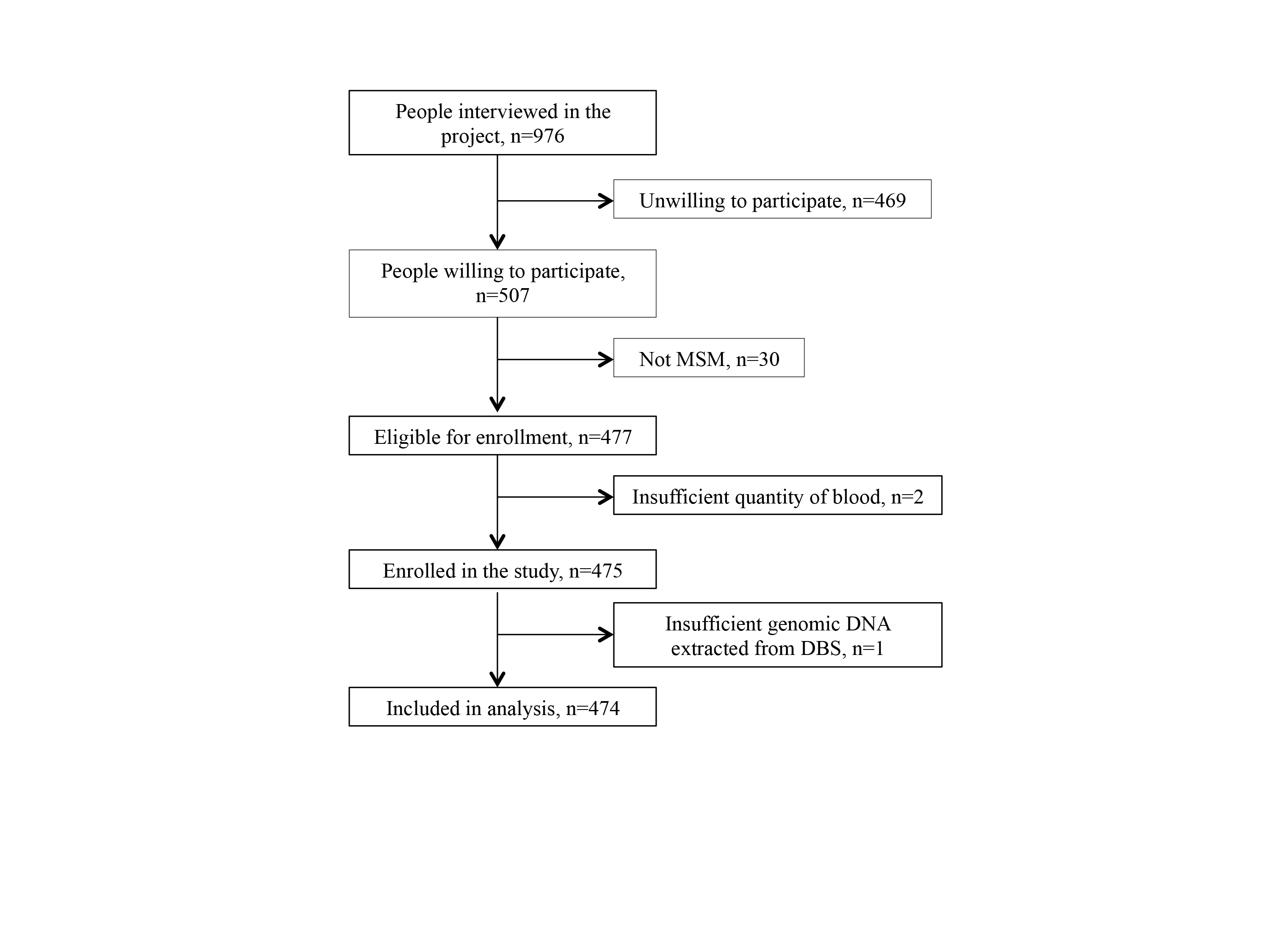

Supplement: S1 Fig — MSM participants screened, enrolled and included in the Hong Kong analysis, March 2010 to February 2011. (TIF) [file pone.0125715.s001.tif]
